# Supplementary material for: Astrocyte-oligodendrocyte interaction regulates central nervous system regeneration
Source: Nat Commun. 2023 Jun 8;14:3372. doi: 10.1038/s41467-023-39046-8 (PMC10250470; doi:10.1038/s41467-023-39046-8)
Supplement: Supplementary file 1 — Supplementary Information [file 41467_2023_39046_MOESM1_ESM.pdf]

Molina-Gonzalez et al:

Astrocyte-Oligodendrocyte interaction regulates central nervous system regeneration

Supplementary Data Table of Contents

Supplementary Data Figures & Legends 1-18

Supplementary Data Table 1

Supplementary data 1 Legend

Supplementary Figure 1. Reactivity of astrocytes during remyelination.

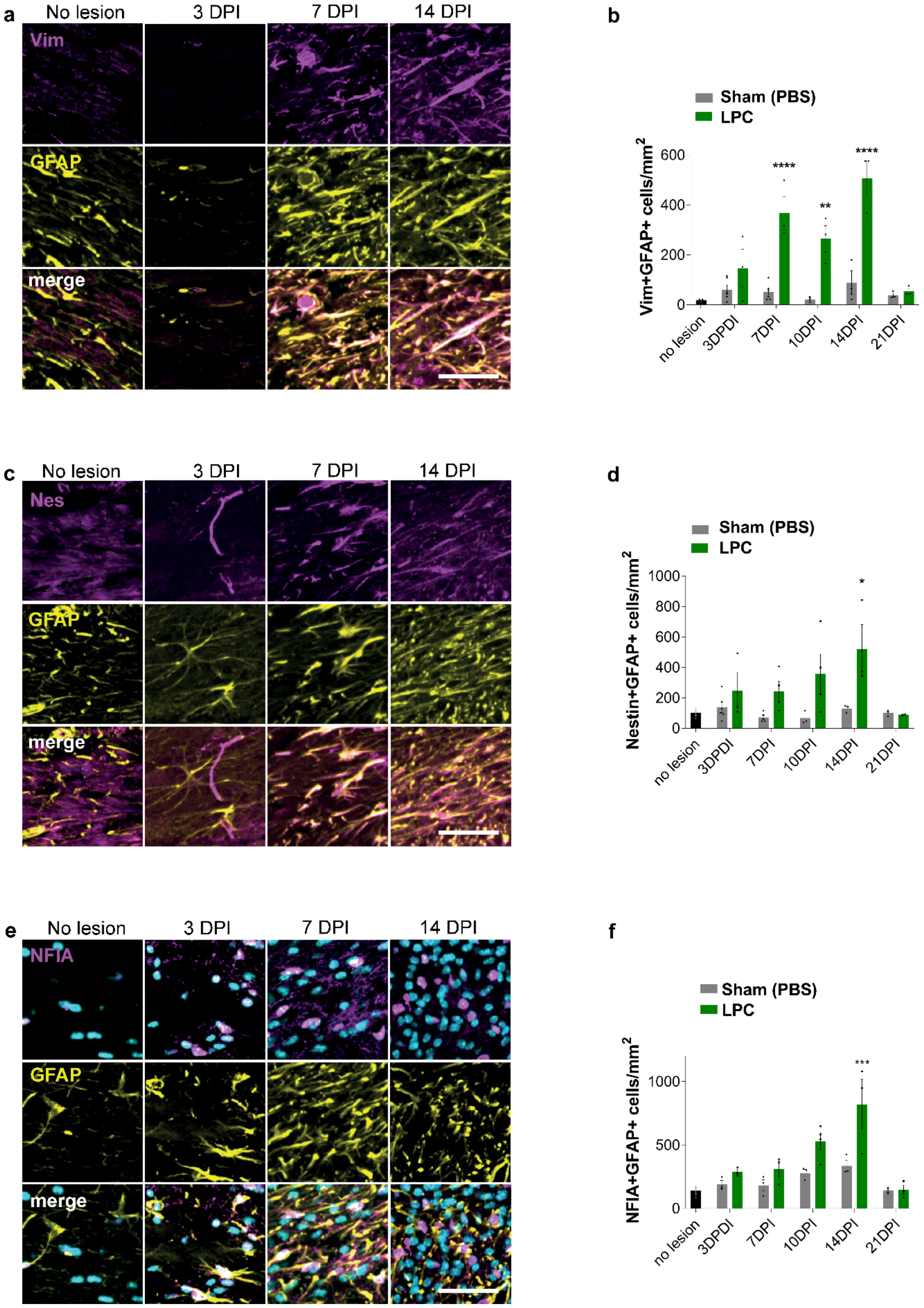

a. Staining for GFAP (yellow) and vimentin (Vim) (magenta) in no lesion control and at 3, 7, and 14 DPI. Scale bar, 50 µm.
b. Vimentin (Vim)+ GFAP+ cells/mm^2 ± s.e.m. in no lesion and Sham (PBS) controls and at 3, 7, 10, 14, and 21 DPI. Two-way ANOVA and Sidak's multiple comparisons test between sham and LPC conditions, adjusted *P* values <0.0001 7 DPI, 0.0017 10 DPI, <0.0001 14 DPI. ANOVA summary *P*<0.0001 for time and condition, F=11.51 and 74.90. n=3 mice/group (LPC 3, 7, 10, 14, 21 DPI; Sham 10, 14, 21 DPI), n=6 mice/group (Sham 3, 7 DPI).
c. Staining for GFAP (yellow) and nestin (Nes; magenta) in no lesion control and at 3, 7, and 14 DPI. Scale bar, 50 µm.
d. Nestin+ GFAP+ cells/mm^2 ± s.e.m. in no lesion and Sham (PBS) controls and at 3, 7, 10, 14, and 21 DPI. Two-way ANOVA and Sidak's multiple comparisons test between sham and LPC conditions, adjusted *P* value 0.0131 14 DPI. ANOVA summary *P*=0.0008 for condition, F=14.50. n=3 mice/group (no lesion, LPC 3, 14; Sham 10, 14, 21 DPI), n=4 mice/group (LPC 7, 10 DPI), n=5 mice/group (Sham 7 DPI), n=6 mice/group (Sham 3 DPI), n=2 mice/group (21 DPI LPC).
e. Staining for GFAP (yellow) and NFIA (magenta) in no-lesion control and at 3, 7, and 14 DPI. Hoechst indicates nuclei in cyan. Scale bar, 50 µm.
f. NFIA+ GFAP+ cells/mm^2 ± s.e.m. in no-lesion and Sham (PBS) controls and at 3, 7, 10, 14, and 21 DPI. Two-way ANOVA and Sidak's multiple comparisons test between sham and LPC conditions, adjusted *P* value 0.0008 14 DPI. ANOVA summary *P*=0.0005, F=17.09 for condition, *P*<0.0001, F=10.42 for time. n=3 mice/group (no lesion, LPC 3, 7, 14, 21 DPI; Sham 3, 7, 10, 14, 21 DPI), n=4 (LPC 10 DPI).
Source data is provided with this paper.

Supplementary Figure 2. Astrocyte numbers and proliferation during remyelination.

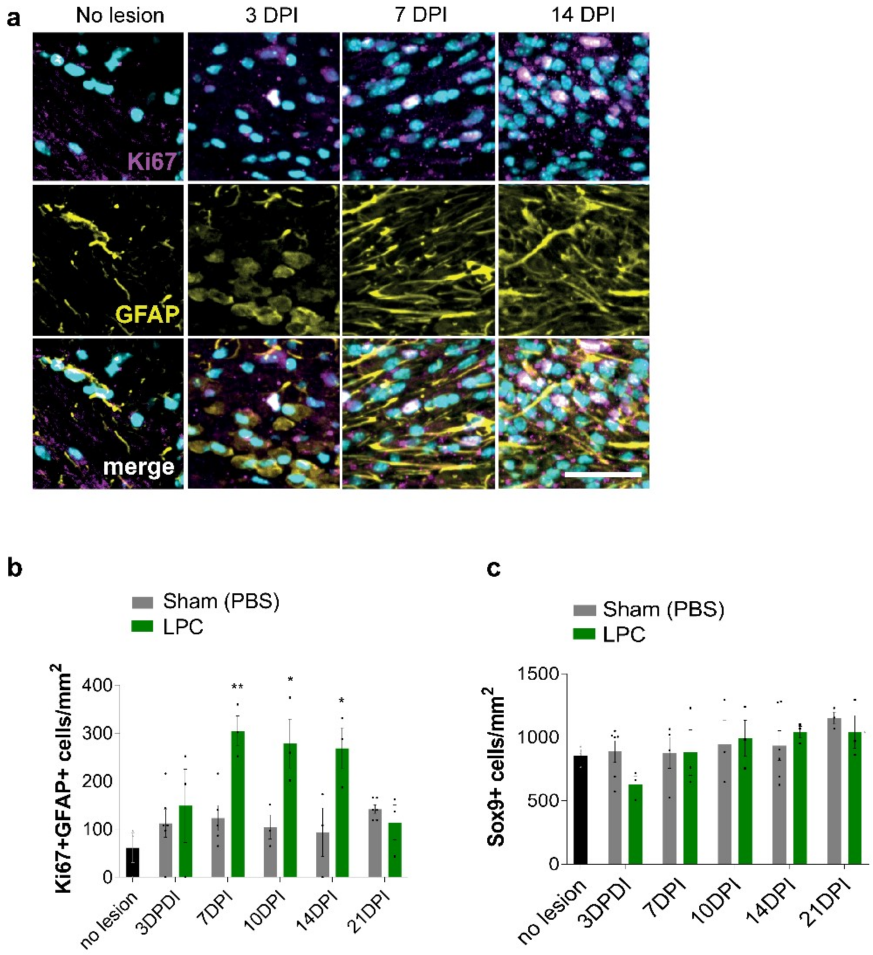

a. Proliferative astrocytes identified with Ki67 (magenta) and GFAP+ (yellow) in no lesion control and at 3, 7, and 14 DPI. Hoechst indicates nuclei in cyan. Scale bar, 100 µm.

b. Mean Ki67+GFAP+ cells/mm^2 ± s.e.m. in Sham (PBS) and no lesion control and at 3, 7, 10, 14, and 21 DPI. Two-way ANOVA with Sidak's multiple comparisons test between Sham and LPC, adjusted *P* values 0.0063 7 DPI, 0.0232 10 DPI, 0.0221 14 DPI. ANOVA summary $P<0.0001$ for Condition. n=3 mice/group (no lesion, LPC 3, 7, 10, 14, 21 DPI; Sham 10, 14 DPI), n=5 mice/group (Sham 7 DPI), n=6 mice/group (Sham 3, 21 DPI).

c. Mean SOX9+ astrocytes/mm^2 ± s.e.m. in Sham (PBS) and no lesion control and at 3, 7, 10, 14, and 21 DPI. Two-way ANOVA with Sidak's multiple comparisons test between Sham and LPC, not significant. n=3 mice/group (no lesion, LPC 3, 7, 10, 14, 21 DPI; Sham 10, 21 DPI), n=4 mice/group (Sham 7 DPI), n=6 mice/group (Sham 3, 14 DPI).

Source data is provided with this paper.

Supplementary Figure 3. Astrocyte reactivity in the cuprizone model of remyelination.

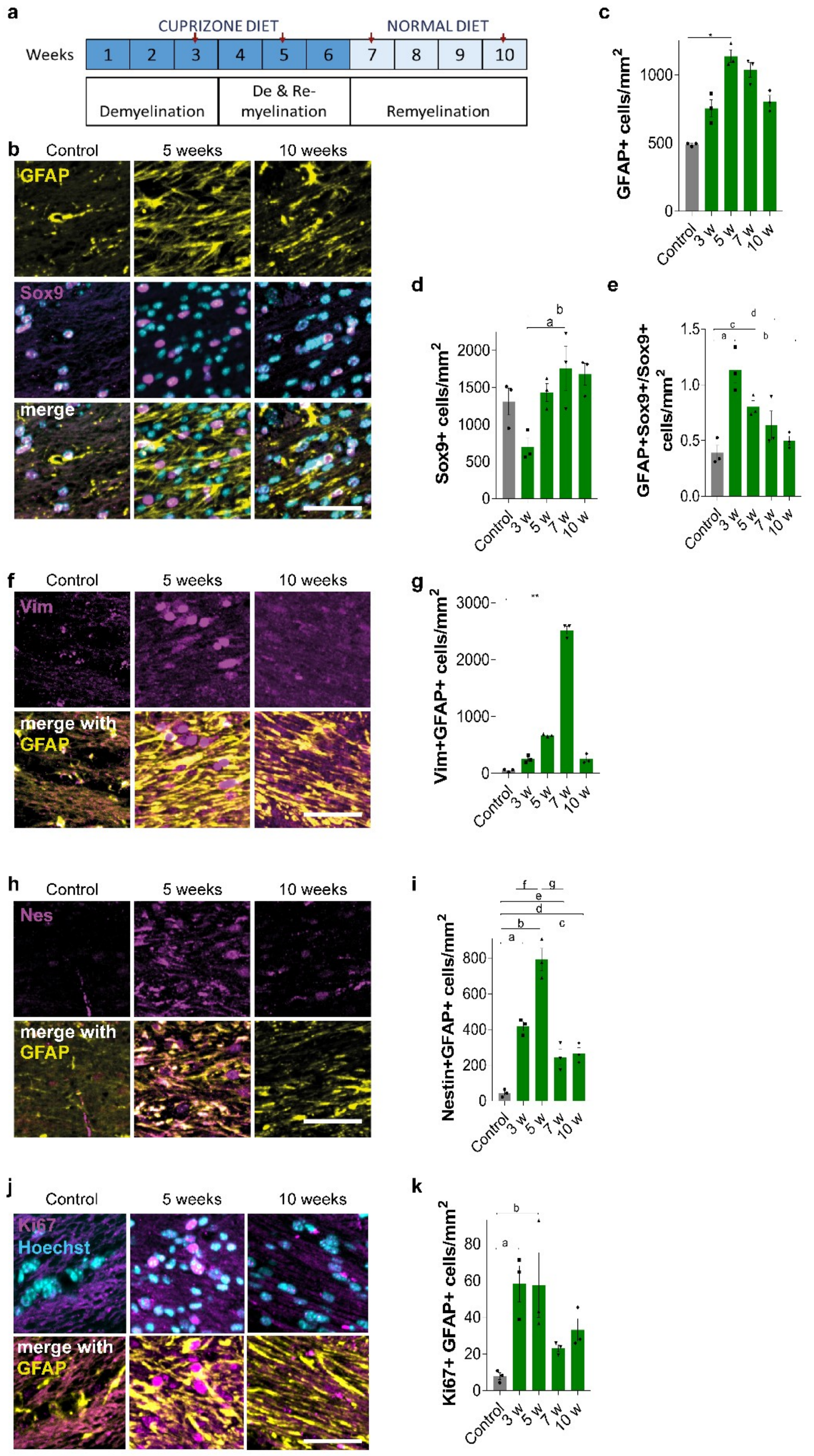

a. Schematic representation of the cuprizone-induced model of demyelination, where red arrowheads indicate key time-points analysed: 3 weeks (demyelination), 5 weeks (concomitant demyelination and early remyelination), 7-10 weeks (late remyelination).

b. Astrocytes stained with GFAP (yellow) and SOX9 (magenta) in no lesion control and at 5 and 10 weeks. Hoechst indicates nuclei in cyan. Scale bar, 50 µm.

c. Mean GFAP+ cells/mm^2 ± s.e.m. in control and at 3, 5, 7, and 10 weeks (w). Kruskal-Wallis test with Dunn's multiple comparisons test, P= 0.0189. ANOVA summary P=0.0001. n=3 mice/group.

d. Mean SOX9+ cells/mm^2 ± s.e.m. in control and at 3, 5, 7, and 10 w. One-way ANOVA with Tukey's multiple comparison test, ^{a}P= 0.0152, ^{b}P= 0.0242. n=3 mice/group. ANOVA summary P=0.0160, F=5.178.

e. Mean GFAP+SOX9+/SOX9+ cell ratio ± s.e.m. in control and at 3, 5, 7, and 10 w. One-way ANOVA with Tukey's multiple comparisons test, ^{a}P=0.0010, ^{b}P=0.0033, ^{c}P=0.0469, ^{d}P=0.0165. ANOVA summary P=0.0011, F=10.99. n=3 mice/group.

f. Astrocytes stained with GFAP (yellow) and Vim (magenta) in no lesion control and at 5 and 10 w. Scale bar, 50 µm.

g. Mean Vim+GFAP+ cells/mm^2 ± s.e.m.. in control and at 3, 5, 7, and 10 w. Kruskal-Wallis test with Dunn's multiple comparisons test, P=0.0099. ANOVA summary P<0.0001. n=3 mice/group.

h. Astrocytes stained with GFAP (yellow) and Nestin (Nes; magenta) in no lesion control and at 5 and 10 w. Scale bar, 50 µm.

i. Mean Nes+GFAP+ cells/mm^2 ± s.e.m.. in control and at 3, 5, 7, and 10 w. One-way ANOVA and Tukey's multiple comparisons test, ^{a}P=0.0004, ^{b}P<0.0001, ^{c}P<0.0001, ^{d}P=0.0160, ^{e}P=0.0291, ^{f}P=0.0004, ^{g}P<0.0001. ANOVA summary P<0.0001 F=50.85. n=3 mice/group.

j. Proliferative astrocytes stained with GFAP (yellow) and Ki67 (magenta) in no lesion control and at 5 and 10 w. Scale bar, 50 µm.

k. Mean Ki67+GFAP+ cells/mm^2 ± s.e.m.. in control and at 3, 5, 7, and 10 w. One-way ANOVA with Tukey's multiple comparisons test, ^{a}P=0.0252, ^{b}P=0.0274. ANOVA summary P=0.0150, F=5.284. n=3 mice/group.

Source data is provided with this paper.

Supplementary Figure 4. Significantly upregulated and downregulated genes in astrocytes during remyelination.

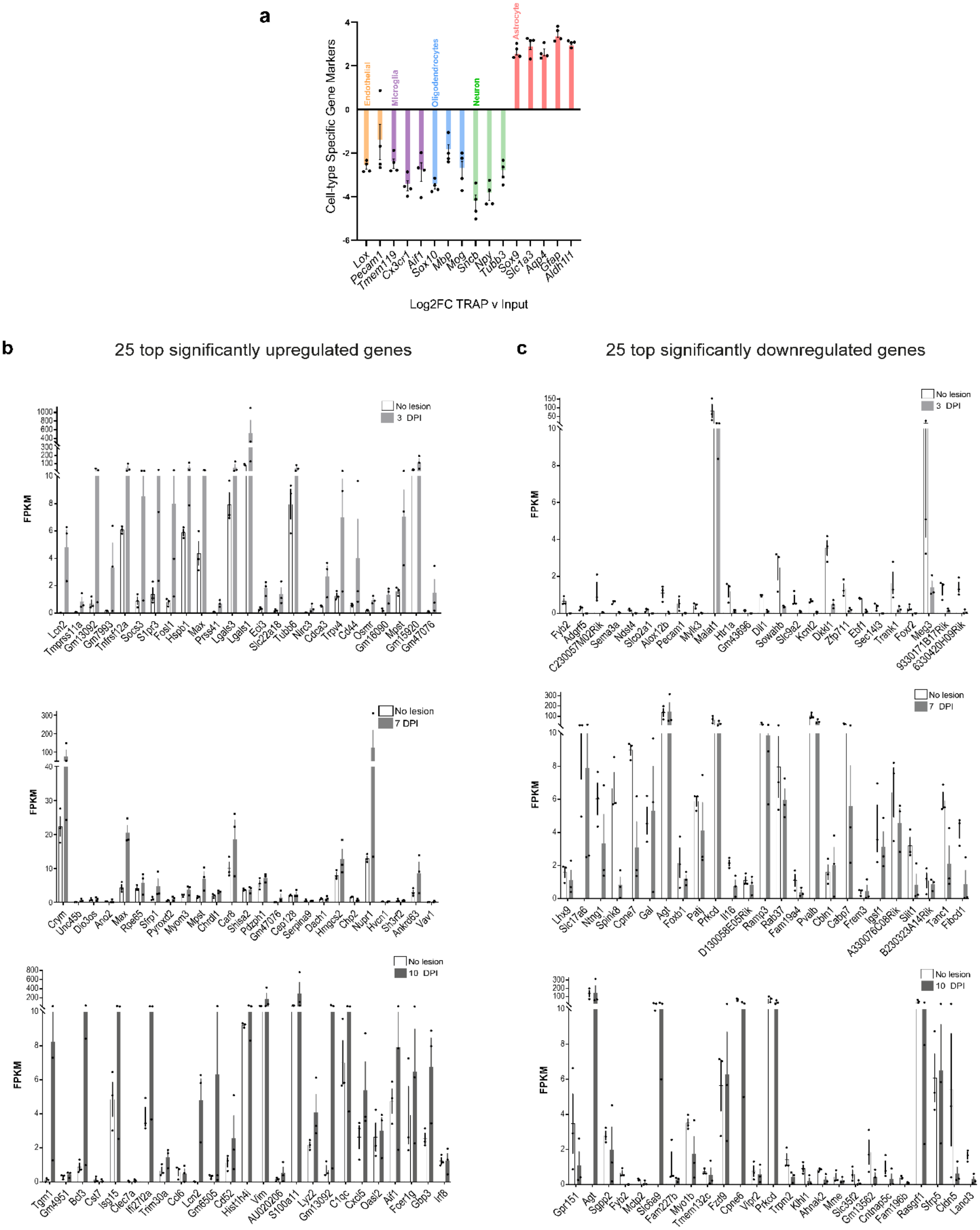

a. Log2 Fold-change (FC) ± s.e.m. in TRAP versus pre-TRAP (input) samples demonstrates enrichment of genes associated with astrocytes (*Aldh1l1, Gfap, Aqp4, Slc1a3, Sox9*) but not those associated with neurons (*Tubb3, Npy, Sncb*), oligodendrocytes (*Mog, Mbp, Sox10*), microglia (*Aif1, Cx3cr1, Tmem119*), or endothelial cells (*Pecam1, Lox*). n=4 mice/condition.
b. Mean FPKM values ± s.e.m. of the 25 top significantly upregulated genes at 3, 7, and 10 DPI. n=3 mice/condition.
c. Mean FPKM values ± s.e.m. of the 25 top significantly downregulated genes at 3, 7, and 10 DPI. n=3 mice/condition.
Source data is provided with this paper.

Supplementary Figure 5. Astrocytes present a mixed inflammatory and neuroprotective signature during remyelination.

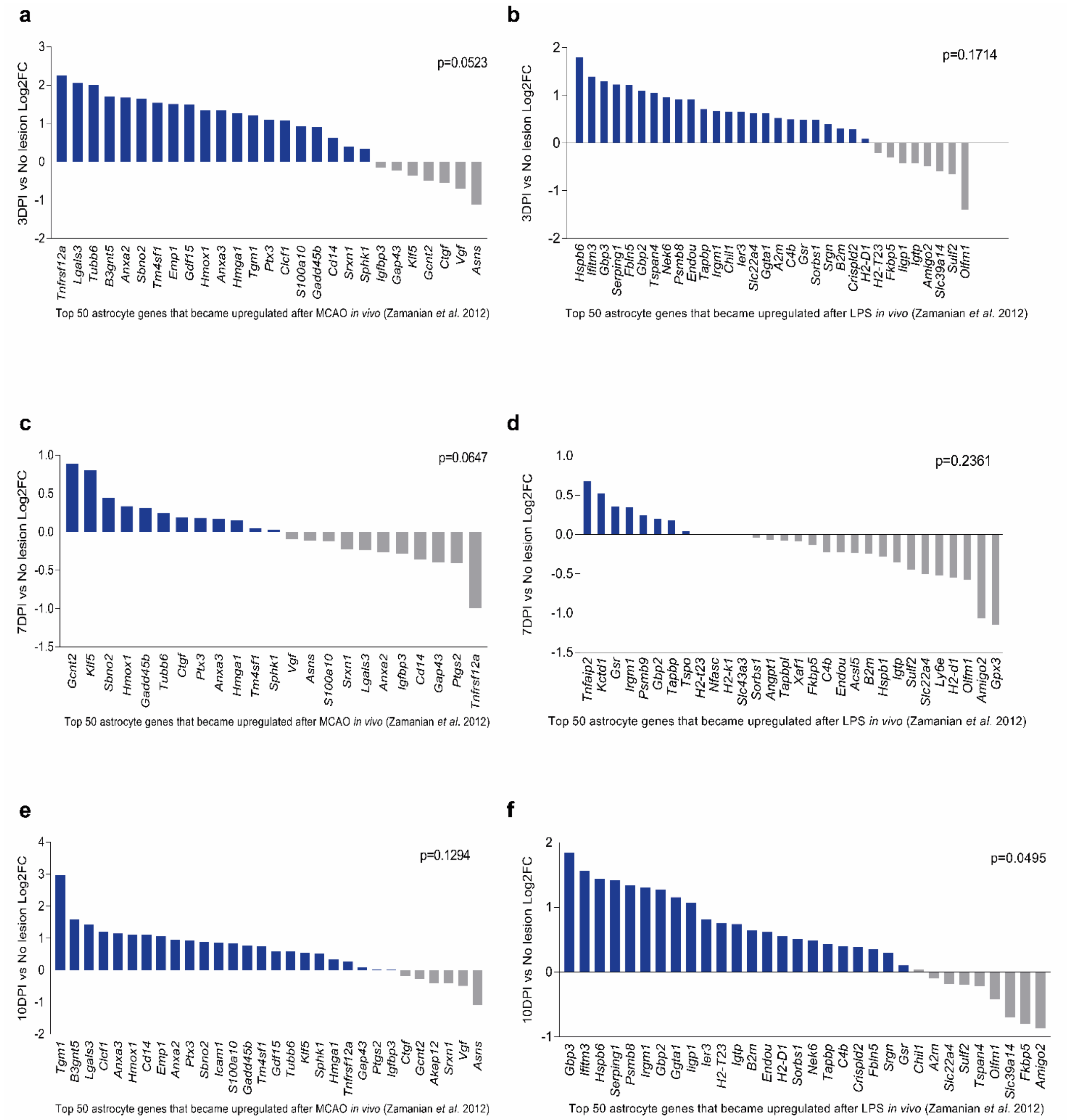

Log2 Fold change (FC) compared to no lesion control at 3 DPI (a, b), 7 DPI (c, d) and 10 DPI (e, f) for the top 50 genes exclusively upregulated in 'A2' astrocytes (after cerebral artery occlusion; a, c, e) and exclusively upregulated in 'A1' astrocytes (after LPS injection; b, d, f). Genes expressed >0.5 FPKM were included in the analysis. One tailed Paired *t*-tests are between the average FPKM compared to no lesion control. n=3 mice/group. Source data is provided with this paper.

Supplementary Figure 6. Gene Ontology pathway analysis of astrocytes during remyelination.

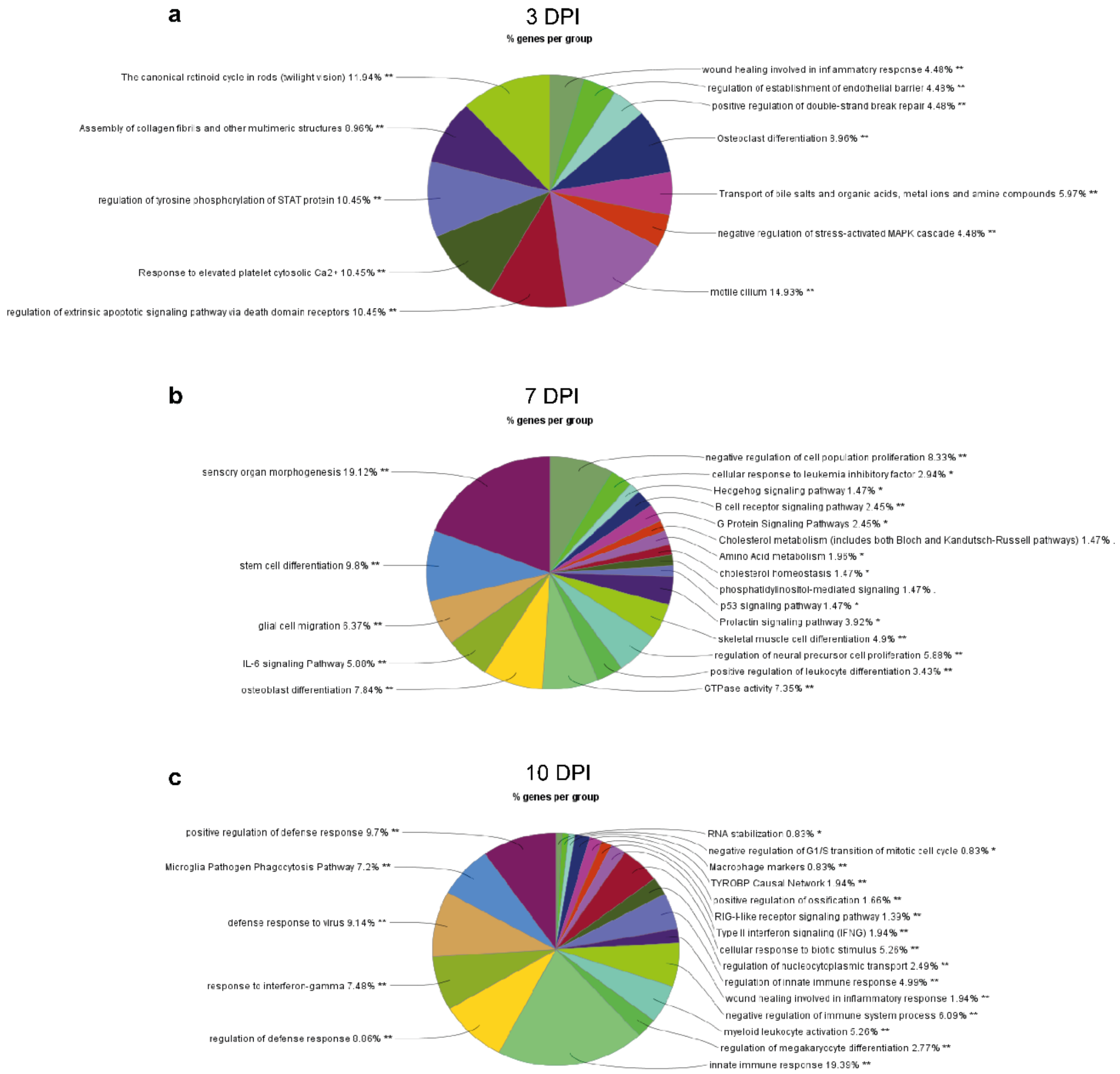

Pie charts showing enriched pathways of the most significantly upregulated Gene Ontology (GO) Terms (adjusted P values <0.05) with a 2-fold change, identified by two-sided hypergeometric test and Bonferroni step down correction, and grouping was based on highest significance and GO-term fusion at 3 DPI (**a**), 7 DPI (**b**), and 10 DPI (**c**). GO terms are grouped using functional grouping (percentage of genes in each GO group) and based on highest significance. Each term was defined with a minimum of 3 genes. Source data is provided with this paper.

Supplementary Figure 7. Nrf2 and cholesterol pathway activation in astrocytes in the cuprizone remyelination model.

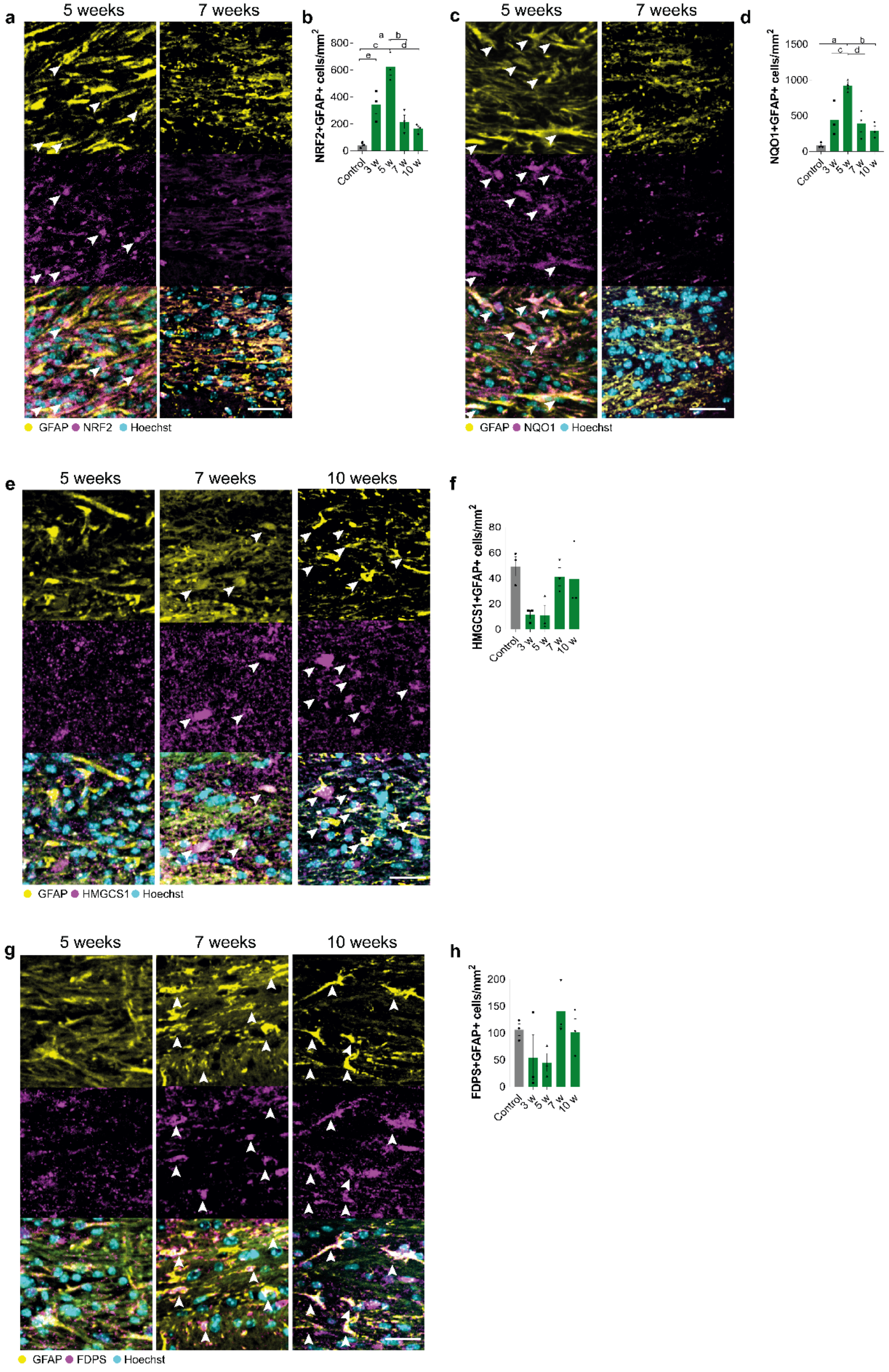

a. Representative images of astrocytes (GFAP+; yellow) expressing NRF2 (magenta) counterstained with Hoechst (cyan), in the corpus callosum of mice during cuprizone-induced demyelination and early remyelination (5 weeks) or during late remyelination on normal diet (7 weeks). Arrows indicate double positive cells. Scale bar, 25μm.

b. Mean densities of NRF2+ GFAP+ astrocytes ± s.e.m. in control mice, during cuprizone-induced demyelination (3, 5 weeks) and during remyelination on normal diet (7, 10 weeks). One-way ANOVA with Tukey's multiple comparisons test; a: *P*=0.0121, b: *P* =0.0008, c: *P* <0.0001, d: *P* =0.0003, e *P*=0.0081. ANOVA summary *P*<0.000,1 F=21.94 n=3 mice/group.

c. Representative images of astrocytes (GFAP+; yellow) expressing Nrf2 target NQO1 (magenta), counterstained with Hoechst (cyan), in the corpus callosum at 5 and 7 weeks of cuprizone paradigm. Arrows indicate double positive cells. Scale bar, 25μm.

d. Mean densities of NQO1+ GFAP+ astrocytes ± s.e.m. in control mice and from 3-10 weeks. One way ANOVA with Tukey's multiple comparisons test; a: *P*=0.0004, b: *P* =0.0035, c: *P* =0.0219, d: *P* =0.0109. ANOVA summary *P*=0.0007, F=12.25. n=3 mice/group.

e. Representative images of astrocytes (GFAP+; yellow) expressing HMGCS1 (magenta), counterstained with Hoechst (cyan), in the corpus callosum at 5 and 7 weeks of cuprizone paradigm. Arrows indicate double positive cells. Scale bar, 25μm.

f. Mean densities of HMGCS1+ GFAP+ astrocytes ± s.e.m. in control mice and from 3-10 weeks. One way ANOVA with Tukey's multiple comparisons, summary *P*=0.0333, F=4.045. n=3 mice/group.

g. Representative images of astrocytes (GFAP+; yellow) expressing FDPS (magenta), counterstained with Hoechst (cyan), in the corpus callosum at 5 and 7 weeks of cuprizone paradigm. Arrows indicate double positive cells. Scale bar, 25μm.

h. Mean densities of FDPS+ GFAP+ astrocytes ± s.e.m. in control mice and from 3-10 weeks. One way ANOVA with Tukey's multiple comparisons test, ANOVA summary *P*=0.1494, F=2.145. n=3 mice/group.
Source data is provided with this paper.

Supplementary Figure 8. Oligodendrocyte lineage cell activation of Nrf2 and cholesterol biosynthesis pathways during remyelination.

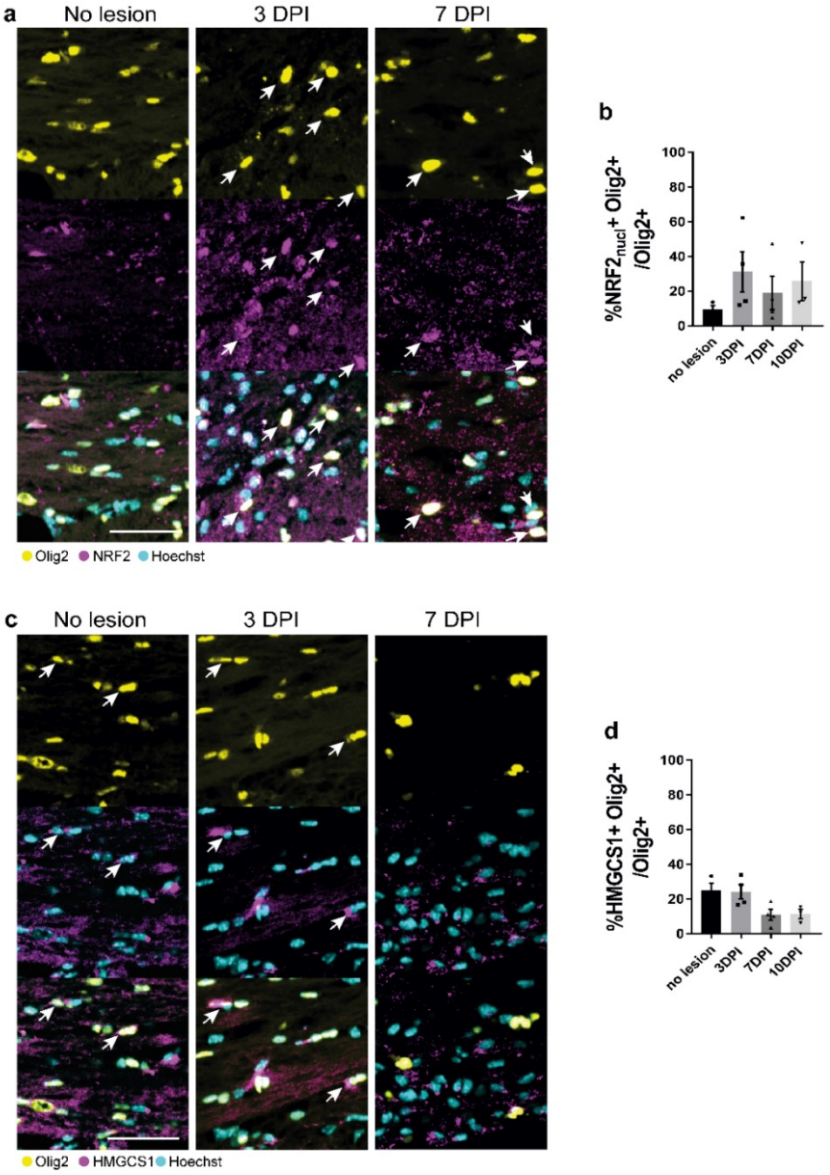

a. Representative images of oligodendrocyte lineage cells (Olig2+; yellow) expressing NRF2 (magenta), counterstained with Hoechst (cyan), in the corpus callosum in non-lesioned control and at 3 and 7 DPI. Arrows indicate double positive cells. Scale bar, 50 μm.

b. Mean percentage of Olig2+ cells expressing activated (nuclear) NRF2 ± s.e.m. in non-lesioned corpus callosum and at 3, 7, and 10 DPI. One-way ANOVA, not significant. ANOVA summary *P*=0.8304, F=5.0470. n=3 mice/group (no lesion, 10 DPI), n=4 mice/group (3, 7 DPI).

c. Representative images of oligodendrocyte lineage cells (Olig2+; yellow) expressing HMGCS1 (magenta), counterstained with Hoechst (cyan), in the corpus callosum in no lesion control and at 3 and 7 DPI. Arrows indicate double positive cells. Scale bar, 50 μm.

d. Mean percentage of Olig2+ cells expressing HMGCS1 ± s.e.m. in non-lesioned corpus callosum and at 3, 7, and 10 DPI. One-way ANOVA, not significant. ANOVA summary *P*=0.0317, F=4.427. n=3 mice/group (no lesion, 10 DPI), n=4 mice/group (3, 7 DPI). Source data is provided with this paper.

Supplementary Figure 9. Astrocyte responses in GFAP-Nrf2 demyelinated lesions.

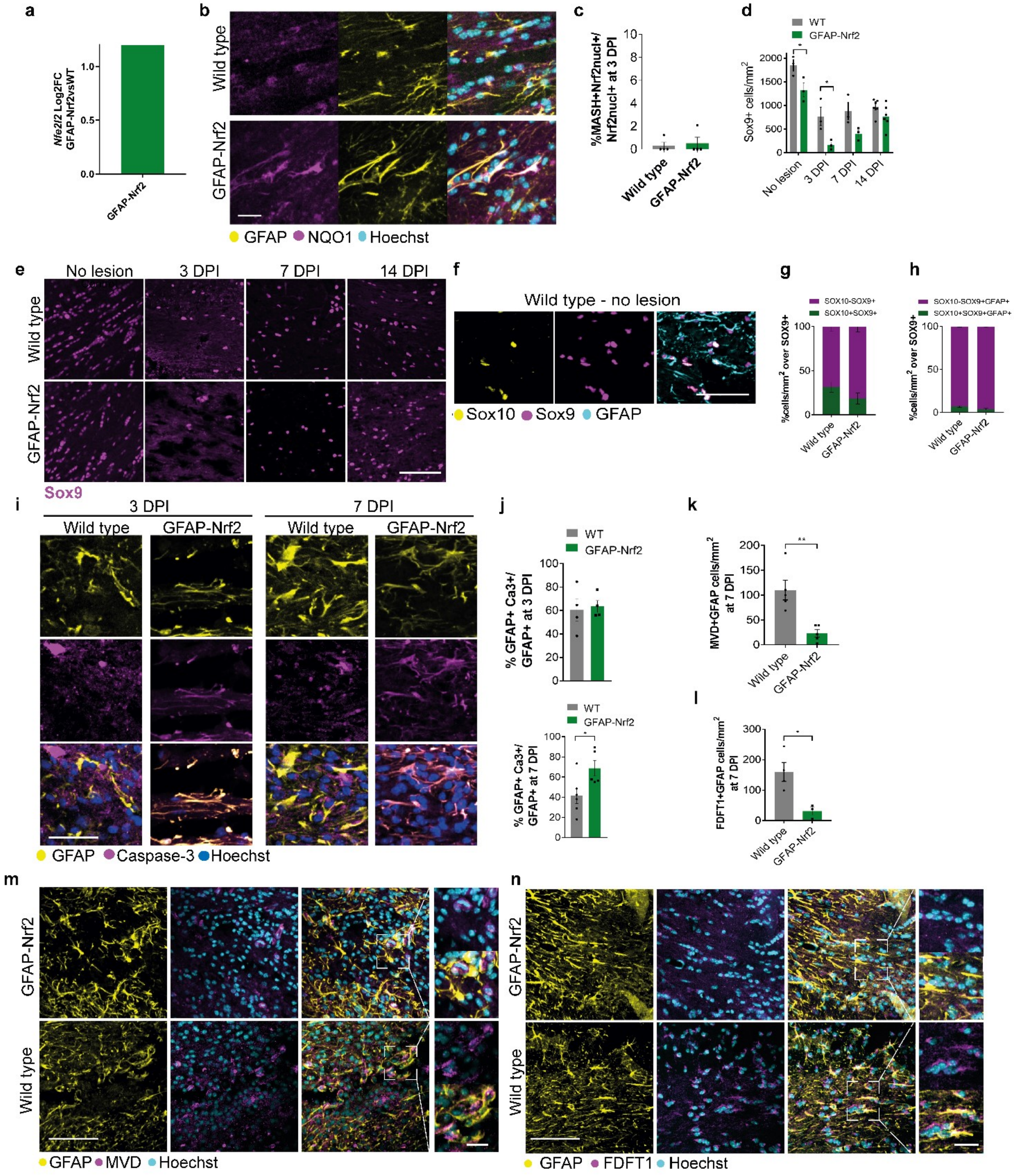

a. Log2 fold change (FC) of the Nrf2 gene *Nfe2l2* in GFAP-Nrf2 astrocytes versus wildtype astrocytes in non-lesioned CNS.

b. Astrocytes (GFAP+; yellow) positive for the Nrf2 target NQO1 (magenta) in non-lesioned corpus callosum of wild type control and GFAP-Nrf2 mice. Hoechst indicates nuclei in cyan. Scale bar, 25 μm.

c. Percentage of nuclear Nrf2+ cells expressing the neural stem cell marker MASH1 ± s.e.m. at 3 DPI in wild type and GFAP-Nrf2 corpus callosum. 2-tailed unpaired Student's t-test, P=0.1127, t=1.857. n=4 mice/group.

d. Mean SOX9+ cells/mm^2 ± s.e.m in wild-type (WT) and GFAP-Nrf2 mice in no lesion control and at 3, 7, and 14 DPI. Two-way ANOVA with Bonferroni correction wild type vs GFAP-Nrf2, a: *P*=0.0427, b: *P*=0.0151. ANOVA summary (Interaction F(3,22)= 1.321, *P*-value=0.2927; Time-point F(3,22)=28.03, *P*-value<0.0001; Genotype F(1,22) = 26.65, *P*-value<0.0001). n=3 mice/ group (Wildtype no lesion, 3 and 7 DPI; GFAP-Nrf2 no lesion, 3 and 7 DPI), n=6 mice/group (Wildtype and GFAP-Nrf2 14 DPI).

e. SOX9+ astrocytes in wild type and GFAP-Nrf2 corpus callosum in no lesion control and at 3, 7 and 14 DPI. Scale bar, 100 μm.

f. Rare examples of GFAP+ (cyan) SOX9+ (magenta) cells expressing SOX10 (yellow). Scale bar, 50 μm.

g. Percentage of SOX9+ cells ± s.e.m. which are SOX10+ (green) or SOX10- (purple) in wild type and GFAP-Nrf2 mice. Two-way ANOVA with Bonferroni correction wild type vs GFAP-Nrf2, not significant. ANOVA summary (Interaction F(1,6)= 3.916, *P*=0.0952; Genotype F(1,6)=0, *P*>0.9999; Cell type F(1,6) = 59.38, *P*-value=0.0003). n=2 mice/group (wildtype), n=3 mice/group (GFAP-Nrf2).

h. Percentage of SOX9+ cells ± s.e.m. which are GFAP+SOX10+ (green) or GFAP+SOX10- (purple) in wildtype and GFAP-Nrf2 mice. Two-way ANOVA with Bonferroni correction wild type vs GFAP-Nrf2, not significant. ANOVA summary (Interaction F(1,6)= 10.38, *P*-value=0.0181; Genotype F(1,6)=0.002923, *P*=0.9586; Cell type F(1,6) = 9153, *P* <0.0001). n=2 mice/group (wildtype), n=3 mice/group (GFAP-Nrf2).

i. GFAP+ astrocytes (yellow) expressing active-Caspase-3+ (magenta) in wild type and GFAP-Nrf2 mouse corpus callosum in no lesion control and at 3, and 7 DPI. Hoechst indicates nuclei in blue. Scale bar, 25 μm.

j. Mean percentage of active-Caspase-3 (Ca3)+ GFAP+ cells over total GFAP+ cells ± s.e.m. in WT and GFAP-Nrf2 mice at 3 and 7 DPI. 2-tailed unpaired Student's *t*-test with Welch's correction, wild type vs GFAP-Nrf2 at 3 DPI *P*=0.7964 t=0.2736. Mann-Whitney test at 7 DPI *P*=0.0303. n=4 mice/group (3DPI), n=6 mice/group (WT 7 DPI), n=5 mice/group (GFAP-Nrf2 7 DPI).

k. Mean MVD+GFAP+ cells/mm^2 ± s.e.m. in WT and GFAP-Nrf2 mice at 7 DPI. 2-tailed unpaired Student's *t*-test with Welch's correction wild type vs GFAP-Nrf2, *P*=0.0087 t=4.137. n=5 mice/condition.

l. Mean FDFT1+GFAP+ cells/mm^2 ± s.e.m. in WT and GFAP-Nrf2 mice at 7 DPI. 2-tailed unpaired Student's *t*-test with Welch's correction wild type vs GFAP-Nrf2, *P*=0.0196 t=3.815. n= 4 mice/condition (WT), n=3 mice/condition (GFAP-Nrf2).

m. Astrocytes (GFAP+;yellow) positive for MVD (magenta) at 7 DPI, Hoechst in cyan. Scale bar, 100 μm. Magnified view, Scale bar, 25 μm.

n. Astrocytes (GFAP+; yellow) positive for FDFT1 (magenta) at 7 DPI, Hoechst in cyan. Scale bar, 100 μm. Magnified view, Scale bar, 25 μm.

Source data is provided with this paper.

Supplementary Figure 10. Oligodendrocyte and microglial responses in GFAP-Nrf2 demyelinated lesions.

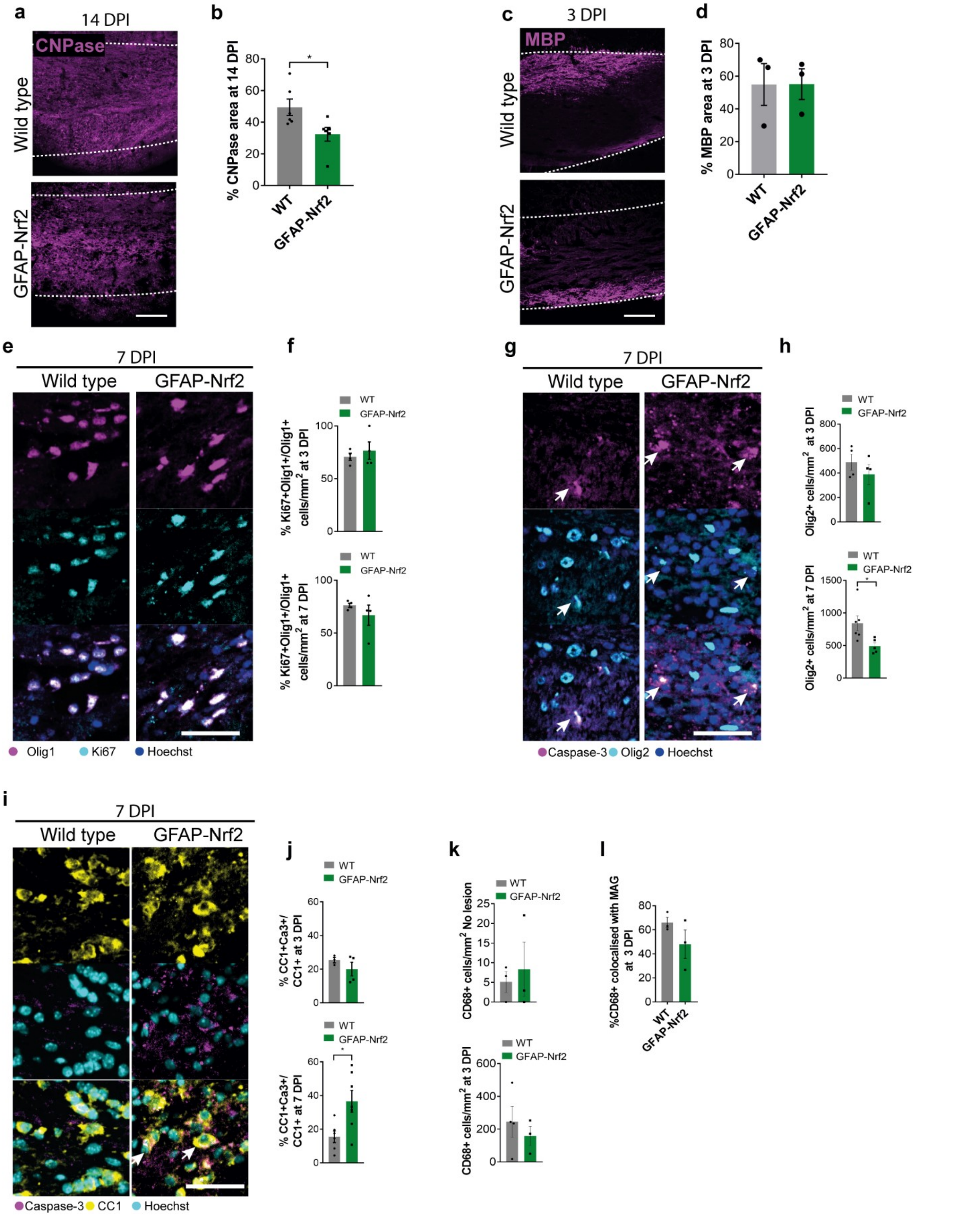

a. CNPase staining (magenta) in the corpus callosum (outlined) in wild type and GFAP-Nrf2 mice at 14 DPI. Scale bar, 100 µm.

b. Percentage of area of corpus callosum with CNPase staining ± s.e.m. in wild type (WT) and GFAP-Nrf2 mice at 14 DPI. 2-tailed unpaired Student's *t*-test with Welch's correction, *P*=0.0299 t=2.53. n=6 mice/group.

c. MBP staining (magenta) in the corpus callosum (outlined) in wild type and GFAP-Nrf2 mice at 3 DPI. Scale bar, 100 µm.

d. Percentage of area of corpus callosum with MBP staining ± s.e.m. in WT and GFAP-Nrf2 mice at 3 DPI. 2-tailed unpaired Student's *t*-test with Welch's correction, *P*=0.9933 t=0.009007, n=3 mice/group.

e. Oligodendrocyte precursors (Olig1+, magenta) which are proliferating (Ki67; cyan) in WT and GFAP-Nrf2 mice at 7 DPI. Hoechst indicates nuclei in blue. Scale bar, 25 µm.

f. Mean percentage of Olig1+ cells which are Ki67+ ± s.e.m. in WT and GFAP-Nrf2 mice at 3 and 7 DPI. 2-tailed unpaired Student's *t*-test with Welch's correction, 3 DPI *P*=0.5547, t=0.6443, 7 DPI *P*=0.405,1 t=0.9545. n=4 mice/group.

g. Oligodendrocyte lineage cells (Olig2+; cyan) which are apoptotic (active Caspase-3+; magenta) in WT and GFAP-Nrf2 mouse corpus callosum at 7 DPI. Hoechst indicates nuclei in blue. Scale bar, 25 µm.

h. Total number of oligodendrocyte lineage cells (Olig2+) cells/mm^2 ± s.e.m. in WT and GFAP-Nrf2 mice at 3 and 7 DPI. 2-tailed unpaired Student's *t*-test with Welch's correction, 3 DPI *P*=0.3854 t=0.9403, 7 DPI *P*=0.0222 t=3.054. n=4 mice/group (Wildtype and GFAP-Nrf2 3 DPI), n=6 mice/group (Wildtype 7 DPI), n=5 mice/group (GFAP-Nrf2 7 DPI).

i. Oligodendrocytes (CC1+; yellow) which are apoptotic (active Caspase-3+; magenta) in WT and GFAP-Nrf2 mouse corpus callosum at 7 DPI. Hoechst indicates nuclei in blue. Scale bar, 25 µm.

j. Mean percentage of oligodendrocytes (CC1+) which are active Caspase-3+ ± s.e.m. in WT and GFAP-Nrf2 mice at 3 and 7 DPI. 2-tailed unpaired Student's *t*-test with Welch's correction, 3 DPI *P*=0.2910, t=1.22, 7 DPI *P*=0.0176 t=2.897. n=3 mice/group (Wildtype 3 DPI), n=4 mice/group (GFAP-Nrf2 3 DPI), n=6 mice/group (Wildtype and GFAP-Nrf2 7 DPI).

k. Mean number of CD68+ microglia/macrophages ± s.e.m. per mm^2 in non-lesioned mice and 3 DPI lesions in WT and GFAP-Nrf2 mice. 2-tailed unpaired Student's *t*-test with Welch's correction, no lesion *P*=0.7004 t=0.4303, 3 DPI *P*=0.4742 t=0.7773. n=3 mice/group.

l. Mean percentage of CD68 area co-localized with myelin associated glycoprotein (MAG) ± s.e.m. at 3 DPI in WT and GFAP-Nrf2 lesions. 2-tailed unpaired Student's *t*-test with Welch's correction, *P*=0.2632 t=1.43. n=3 mice/group.

Source data is provided with this paper.

Supplementary Figure 11. Astrocyte depletion is associated with oligodendrocyte death during remyelination.

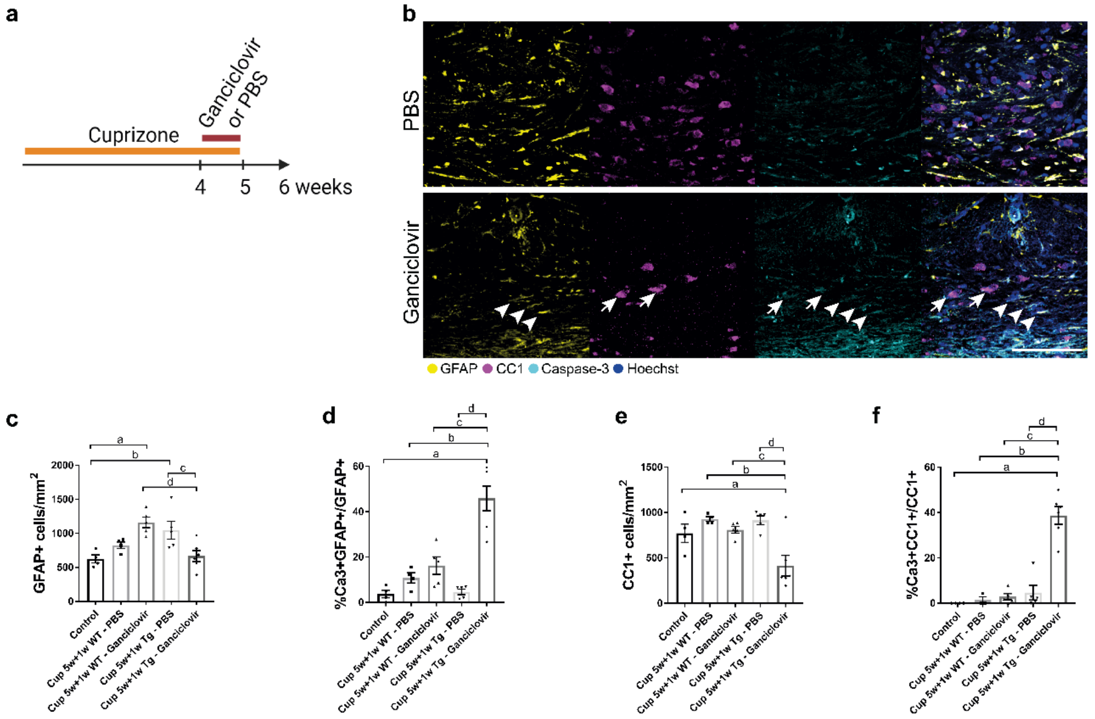

a. Cuprizone diet was provided to wildtype mice or GFAP-thymidine kinase mice for 5 weeks to induce demyelination. Ganciclovir was administered from weeks 4-5 at the onset of remyelination to induce astrocyte death. PBS vehicle administration served as a control. Mice were sacrificed at 6 weeks after 1 week on normal diet.

b. Astrocyte (GFAP+; yellow) and oligodendrocyte (CC1+; magenta) expressing the apoptotic marker active caspase-3 (cyan), and counterstained with Hoechst (blue). Apoptotic astrocytes are indicated with arrowheads, whilst apoptotic oligodendrocytes are indicated with arrows. Scale bar, 50µm.

c. Mean number of GFAP+ astrocytes ± s.e.m. per mm^2 in control diet mice, wildtype (WT) mice fed with cuprizone and treated with PBS or ganciclovir, and transgenic mice (Tg) fed with cuprizone treated with PBS or ganciclovir. One way ANOVA with Tukey's multiple comparisons test, a: P=0.0054, b: P =0.034, c: P =0.0362, d: P =0.0047. ANOVA summary F=6.887 and P-value=0.0013. n=4 mice/group (control and Cup5w + 1w WT-PBS), n=5 mice/group (Cup5w + 1w WT-Ganciclovir, Cup5w + 1w Tg-PBS), n=6 mice/group (Cup5w + 1w Tg- Ganciclovir).

d. Mean percentage of GFAP+ astrocytes positive for active caspase-3 ± s.e.m. in the above conditions. One way ANOVA with Tukey's multiple comparisons test; a-d, P<0.0001. ANOVA summary F=24.10 and P<0.0001. n=4 mice/group (control and Cup5w + 1w WT-PBS), n=5 mice/group (Cup5w + 1w WT-Ganciclovir, Cup5w + 1w Tg-PBS), n=6 mice/group (Cup5w + 1w Tg- Ganciclovir).

e. Mean number of CC1+ oligodendrocytes ± s.e.m. in the above conditions. One way ANOVA with Tukey's multiple comparisons test; a: P=0.0383, b: P =0.002, c: P =0.0117, d: P =0.0013. ANOVA summary F=7.699 and P-value=0.0007. n=4 mice/group (control and Cup5w + 1w WT-PBS), n=5 mice/group (Cup5w + 1w WT-Ganciclovir, Cup5w + 1w Tg-PBS), n=6 mice/group (Cup5w + 1w Tg- Ganciclovir).

f. Mean percentage of CC1+ oligodendrocytes positive for active caspase-3 ± s.e.m. in the above conditions. One way ANOVA with Tukey's multiple comparisons test; a-d P<0.0001. ANOVA summary F=36.73 and P<0.0001. n=3 mice/group (Cup5w + 1w WT-PBS), n=4 mice/group (control), n=5 mice/group (Cup5w + 1w WT-Ganciclovir, Cup5w + 1w Tg-PBS), n=6 mice/group (Cup5w + 1w Tg- Ganciclovir).

Source data is provided with this paper.

Supplementary Figure 12. Oligodendrocyte and microglial responses to CS-6253 treatment in GFAP-Nrf2 mice.

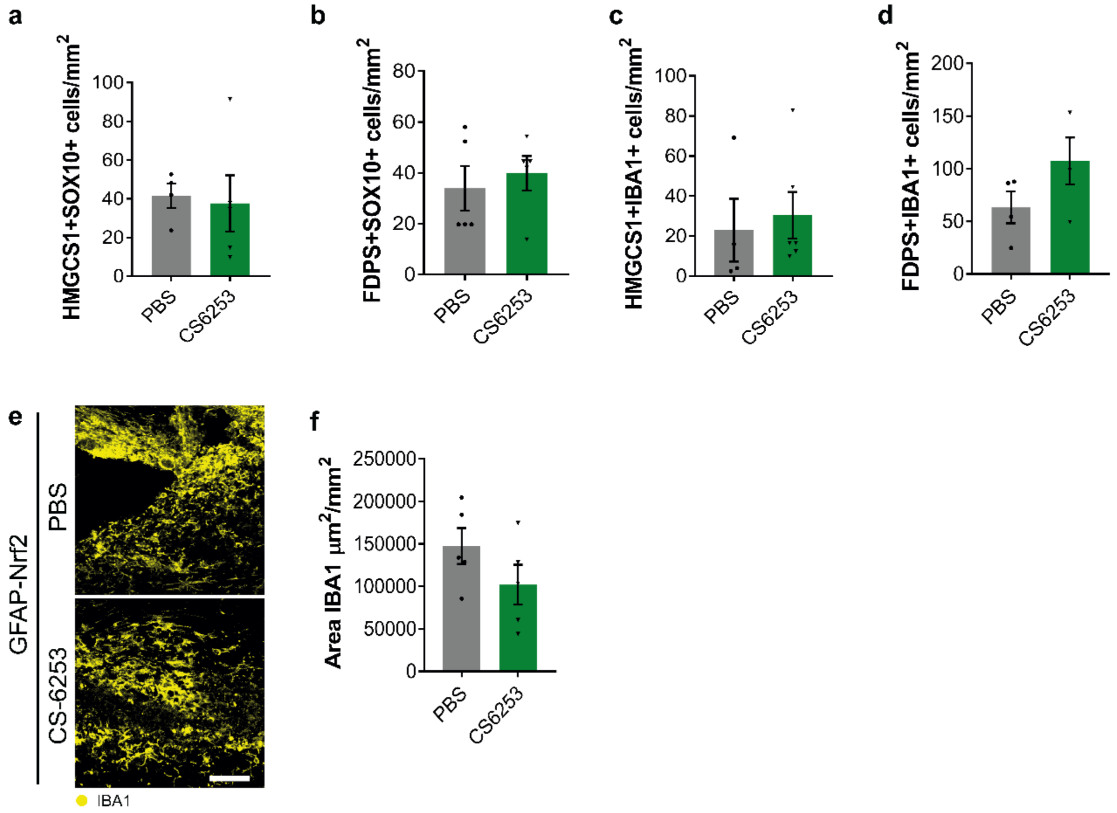

- **a.** Mean number of oligodendrocyte lineage cells (SOX10+) expressing HMGCS1 per mm^2 ± s.e.m. in GFAP-Nrf2 mice treated with PBS or CS-6253. 2-tailed unpaired Student's *t*-test with Welch's correction, P=0.8128 t=0.2486. n=4 mice/group (PBS), n=5 mice/group (CS6253).
- **b.** Mean number of FDPS+SOX10+ cells per mm^2 ± s.e.m. in GFAP-Nrf2 mice treated with PBS or CS-6253. 2-tailed unpaired Student's *t*-test with Welch's correction, P=0.6108 t=0.5308. n=5 mice/group.
- **c.** Mean number of microglia/macrophages (IBA1+) expressing HMGCS1 per mm^2 ± s.e.m. in GFAP-Nrf2 mice treated with PBS or CS-6253. 2-tailed unpaired Student's *t*-test with Welch's correction, P=0.7150 t=0.3826. n=4 mice/group (PBS) and n=6 mice/group (CS6253).
- **d.** Mean number of FDPS+IBA1+ cells ± s.e.m. per mm^2 in GFAP-Nrf2 mice treated with PBS or CS-6253. 2-tailed unpaired Student's *t*-test with Welch's correction, P=0.1587 t=1.642. n=4 mice/group.
- **e.** IBA1 staining in PBS or CS-6253 treated GFAP-Nrf2 mice. Scale bar, 50μm.
- **f.** Mean area of IBA1 staining ($μm^2$) per mm^2 ± s.e.m. in PBS or CS-6253 treated GFAP-Nrf2 mice. 2-tailed unpaired Student's *t*-test with Welch's correction, P=0.1886 t=1.438. n=5 mice/group.

Source data is provided with this paper.

Supplementary Figure 13. Conditional knockout of Nrf2 in astrocytes

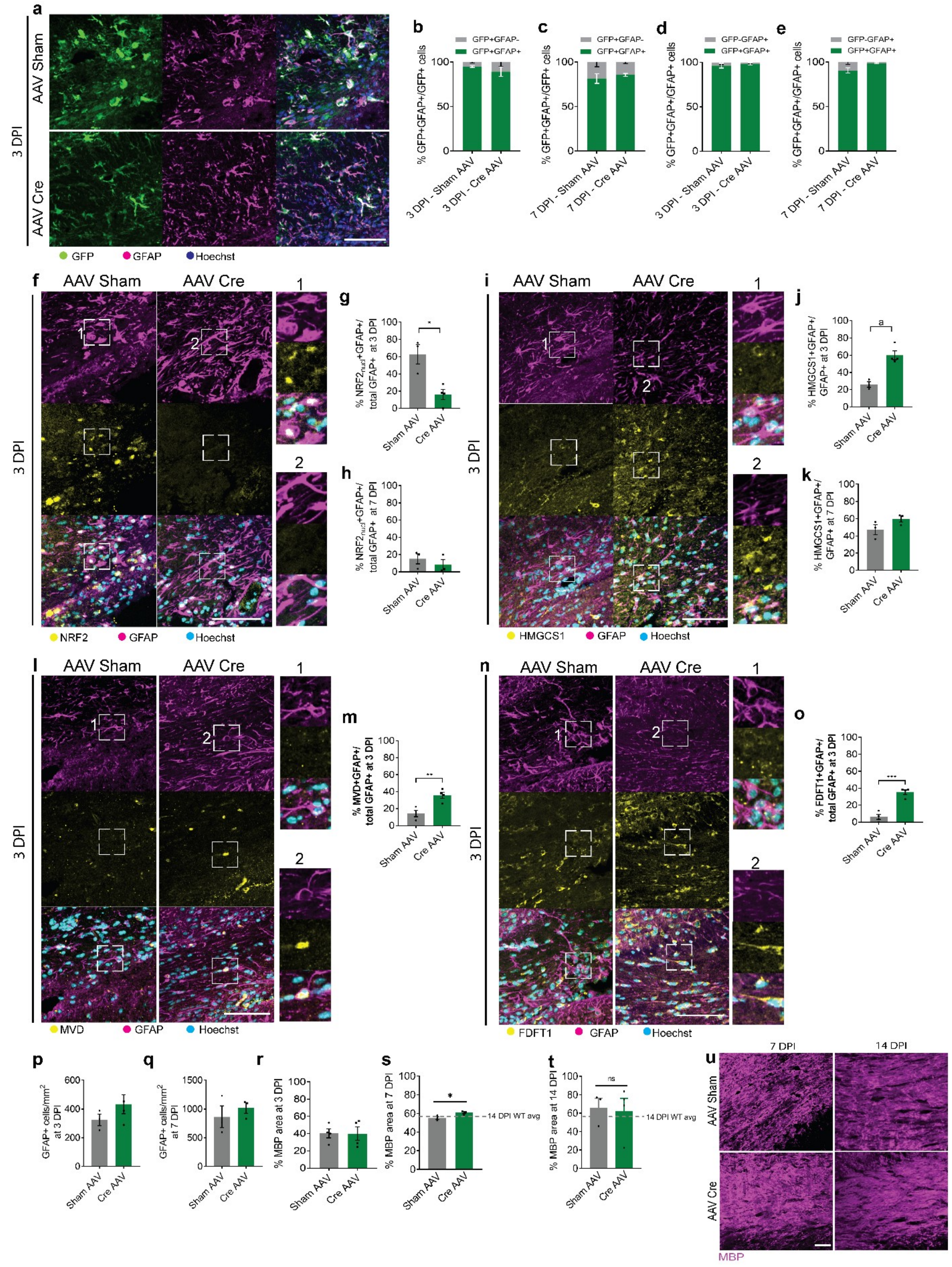

a. GFP expression (green) colocalized with GFAP (magenta) and counterstained with Hoechst (blue), following injection of *Nfe2l2* floxed mice with AAV5-GFAP(0.7)-eGFP-T2A-iCre ('AAV-Cre') or AAV5-GFAP(0.7)-eGFP ('AAV Sham') into the corpus callosum. Scale bar, 100 µm.
b. Specificity of viral transduction indicated as mean percentage of GFP+ signal which was colocalized with GFAP ± s.e.m. at 3 DPI following injection with Sham AAV or Cre AAV. n=4 mice per condition.
c. Specificity of viral transduction indicated as mean percentage of GFP+ signal which was colocalized with GFAP ± s.e.m. at 7 DPI following injection with Sham AAV or Cre AAV. n=3 mice per condition.
d. Efficiency of viral transduction indicated as mean percentage of GFAP+ cells which were GFP+ ± s.e.m. at 3 DPI following injection with Sham AAV or Cre AAV. n=4 mice per condition.
e. Efficiency of viral transduction indicated as mean percentage of GFAP+ cells which were GFP+ ± s.e.m. at 7 DPI following injection with Sham AAV or Cre AAV. n=3 mice per condition.
f. Astrocytes (GFAP+; magenta) expressing nuclear Nrf2 (yellow) and counterstained with Hoechst (blue) in AAV Sham and AAV Cre mice at 3 DPI. Magnifications indicated for AAV Sham (1) and AAV Cre (2). Scale bar, 100 µm.
g. Mean percentage of GFAP+ cells with nuclear Nrf2 at 3 DPI in Sham AAV and Cre AAV mice ± s.e.m. 2-tailed unpaired Student's t-test, ^{a}P =0.0104 t=5.186. n=3 mice/condition (sham), n=4 mice per condition (Cre).
h. Mean percentage of GFAP+ cells with nuclear Nrf2 at 7 DPI in Sham AAV and Cre AAV mice ± s.e.m.. 2-tailed unpaired Student's test, *P*=0.4488, t=0.8388. n=3 mice per condition.
i. Astrocytes (GFAP+; magenta) expressing HMGCS1 (yellow) and counterstained with Hoechst (blue) in AAV Sham and AAV Cre mice at 3 DPI. Magnifications indicated for AAV Sham (1) and AAV Cre (2). Scale bar, 100 µm.
j. Mean percentage of GFAP+ cells expressing HMGCS1 at 3 DPI in Sham AAV and Cre AAV mice ± s.e.m.. 2-tailed unpaired Student's t-test with Welch's correction, ^{a}P =0.0028, t=5.653. n=3 mice/group (Sham AAV), n=4 mice/group (Cre AAV).
k. Mean percentage of GFAP+ cells expressing HMGCS1 at 7 DPI in Sham AAV and Cre AAV mice ± s.e.m.. 2-tailed unpaired Student's test, *P*=0.3226 t=1.125. n=3 mice per condition.
l. Astrocytes (GFAP+; magenta) expressing MVD (yellow) and counterstained with Hoechst (blue) in AAV Sham and AAV Cre mice at 3 DPI. Magnifications indicated for AAV Sham (1) and AAV Cre (2). Scale bar, 100 µm.
m. Mean percentage of GFAP+ cells expressing MVD at 7 DPI in Sham AAV and Cre AAV mice ± s.e.m.. 2-tailed unpaired Student's *t*-test, *P*=0.0052 t=4.274. n=4 mice per condition.
n. Astrocytes (GFAP+; magenta) expressing FDFT1 (yellow) and counterstained with Hoechst (blue) in AAV Sham and AAV Cre mice at 3 DPI. Magnifications indicated for AAV Sham (1) and AAV Cre (2). Scale bar, 100 µm.
o. Mean percentage of GFAP+ cells expressing FDFT1 at 7 DPI in Sham AAV and Cre AAV mice ± s.e.m.. 2-tailed unpaired Student's *t*-test, *P*=0.0003 t=7.337. n=3 mice/condition (sham), n=4 mice per condition (Cre).
p. Mean astrocyte numbers (GFAP+) in AAV Sham and AAV Cre mice at 3 DPI ± s.e.m.. 2-tailed unpaired Student's test, *P*=0.2192 t=1.42. n=3 mice per condition (sham), n=4 mice/condition (Cre).
q. Mean astrocyte numbers (GFAP+) in AAV Sham and AAV Cre mice at 7 DPI ± s.e.m..2-tailed unpaired Student's test, *P*=0.5200 t=0.7291. n=3 mice per condition.
r. Mean percentage of MBP area at 3 DPI (indicating demyelination) in Sham AAV and AAV Cre mice ± s.e.m.. 2-tailed unpaired Student's test, *P*=0.9759 t=0.03147. n=4 mice per condition.
s. Mean percentage of MBP area (indicating remyelination) in AAV Sham and AAV Cre mice at 7 DPI ± s.e.m.. 2-tailed unpaired Student's t-test, ^{a}P =0.0366 t=3.09. n=3 mice per condition. Average value in 14 DPI wildtype remyelinated lesions indicated as dotted line.
t. Mean percentage of MBP area (indicating remyelination) in AAV Sham and AAV Cre mice at 14 DPI ± s.e.m.. 2-tailed unpaired Student's t-test, *P* =0.8493 t=0.2001. n=3 mice per condition (sham), n=4 mice/condition (Cre). Average value in 14 DPI wildtype remyelinated lesions indicated as dotted line.
u. Myelin basic protein (MBP) staining (magenta) at 7 and 14 DPI in AAV Sham and AAV Cre. Scale bar, 25 µm.
Source data is provided with this paper.

Supplementary Figure 14. Primary astrocyte uptake of fluorescent cholesterol analogue.

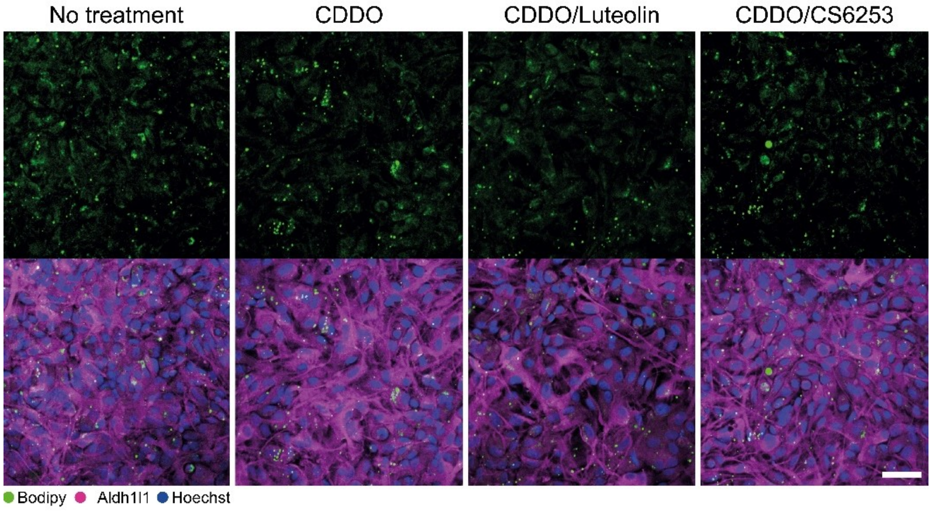

Primary astrocytes (Aldh1l1+; magenta) having taken up Bodipy-FL-C12 fluorescent cholesterol analogue (green), counterstained with Hoechst (blue). Astrocytes were either untreated, or treated with $CDDO^{TFEA}$ to induce Nrf2 activation, then either untreated or treated with the Nrf2 inhibitor Luteolin or the cholesterol biosynthesis pathway inducer/efflux stimulator CS-6253. Scale bar, 50 µm.

Supplementary Figure 15. Astrocyte responses in multiple sclerosis lesions.

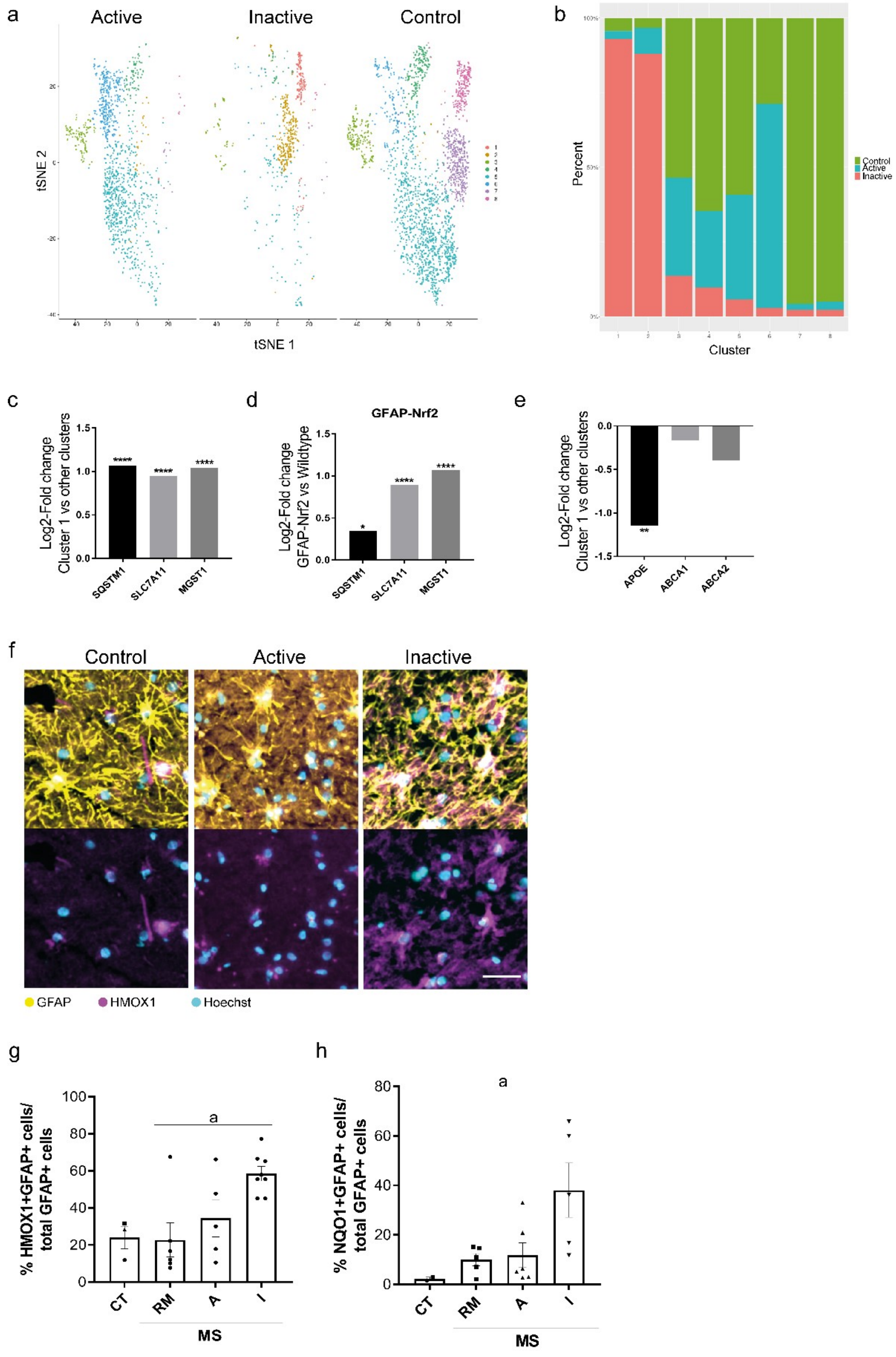

- **a.** tSNE plot of single nuclei sequencing of astrocytes from control white matter, or active and inactive multiple sclerosis (MS) lesions. Legend indicates cluster colour assignments.
- **b.** Percent frequency distribution of astrocyte clusters among control, and active and inactive MS lesions.
- **c.** Log2 Fold Change of expression of Nrf2-target genes in Cluster 1 over other clusters. $P=7.83 \times 10^{-8}$, 1.91×10^{-9}, $P=1.26 \times 10^{-11}$. n=12 MS cases, n=9 control cases. Non-parametric Wilcoxon rank sum test.
- **d.** GFAP-Nrf2 mouse astrocyte expression of Nrf2 target genes upregulated in human Cluster 1 astrocytes. Represented as Log2-fold change vs wild type control astrocytes. DESeq2 and Benjamini-Hochberg test, adjusted *P* values 0.0137, 5.88×10^{-37}, 1.84×10^{-16}. n=3 mice per condition.
- **e.** Log2 Fold Change of expression of cholesterol export genes in Cluster 1 over other clusters. $^{a}P=1.97 \times 10^{-3}$. n=12 MS cases, n=9 control cases. Non-parametric Wilcoxon rank sum test.
- **f.** Astrocytes (GFAP+; yellow) expressing HMOX1 (magenta) counterstained with Hoechst (cyan) in control human white matter, and active and inactive multiple sclerosis lesions. Scale bar, 25 µm.
- **g.** Mean percentage of GFAP+ cells expressing HMOX1 ± s.e.m. in control (CT; n=3), fully remyelinated (RM; n=6), active (A; n=5) and inactive (I; n=8) multiple sclerosis (MS) lesions. Kruskal-Wallis and Dunn's multiple comparisons test, $^{a}P=0.0344$.
- **h.** Mean percentage of GFAP+ cells expressing NQO1 ± s.e.m. in CT (n=2), RM (n=5), A (n=6), and I (n=5) MS lesions. Kruskal-Wallis and Dunn's multiple comparisons test, $^{a}P=0.0353$.
  Source data is provided with this paper.

Supplementary Figure 16. Responses to Luteolin treatment.

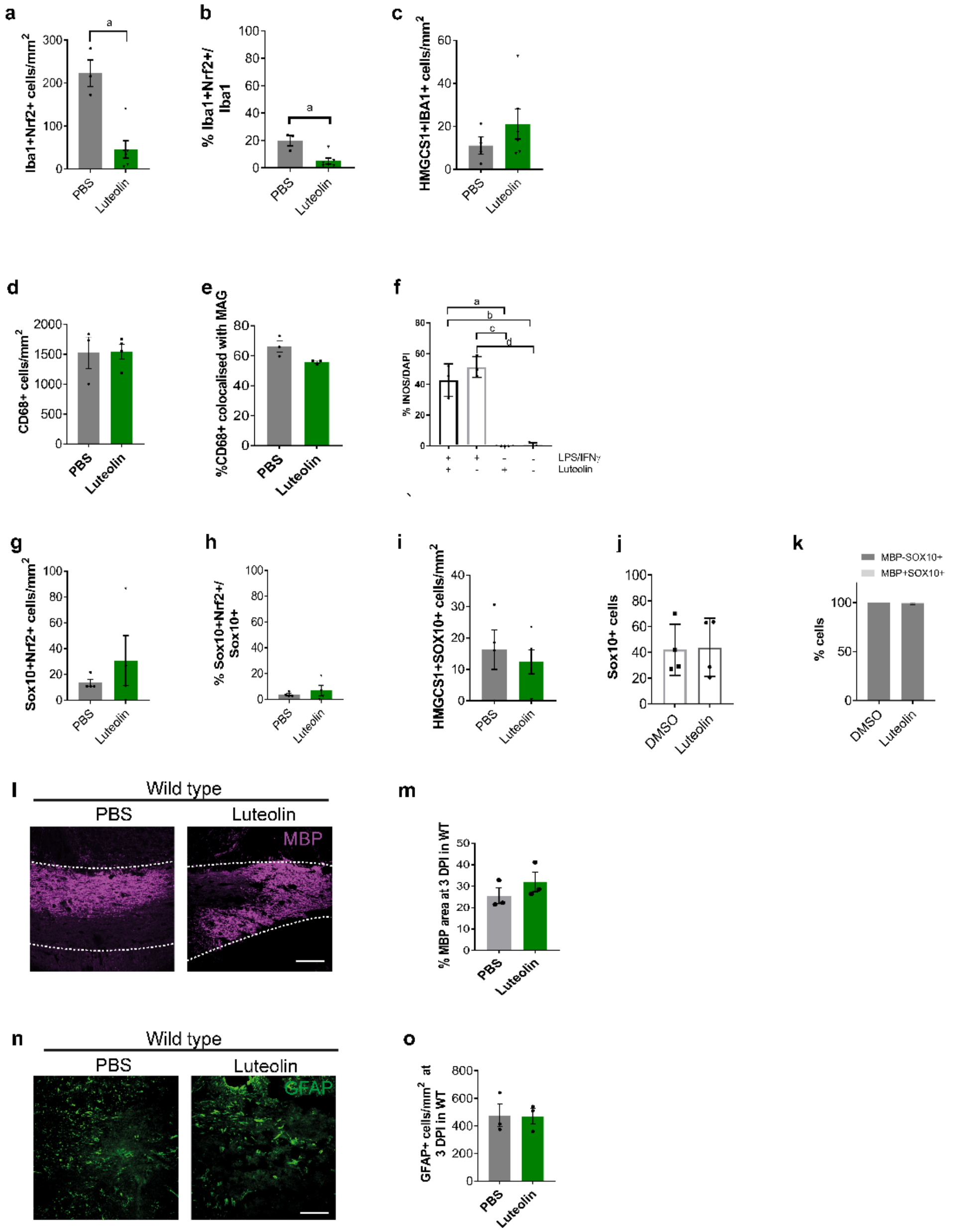

- **a.** Mean number of microglia/macrophages (IBA1+) expressing Nrf2 per mm^2 ± s.e.m. in PBS or Luteolin treated GFAP-Nrf2 mice. 2-tailed Mann-Whitney test, [a]*P*=0.0238. n=3 mice/group (PBS), n=6 mice/group (Luteolin).
- **b.** Percentage of IBA1+ cells expressing Nrf2 per mm^2 ± s.e.m. in PBS or Luteolin treated GFAP-Nrf2 mice. 2-tailed Mann-Whitney test, [a]*P*=0.0476. n=3 mice/group (PBS), n=6 mice/group (Luteolin).
- **c.** Mean number of IBA1+ HMGCS1+ cells per mm^2 ± s.e.m. in PBS or Luteolin treated GFAP-Nrf2 mice. 2-tailed unpaired Student's t-test, *P*=0.2498, t=1.248. n=4 mice/group (PBS), n=6 mice/group (Luteolin).
- **d.** Mean number of microglia/macrophages (CD68+) per mm^2 ± s.e.m. in PBS or Luteolin treated GFAP-Nrf2 mice. 2-tailed unpaired Student's t-test, *P*=0.9544 t=0.06005. n=3 mice/group (PBS), n=4 mice/group (Luteolin).
- **e.** Percentage of CD68 area co-localized with myelin associated glycoprotein (MAG) per mm^2 ± s.e.m. in PBS or Luteolin treated GFAP-Nrf2 mice. 2-tailed unpaired Student's t-test, *P*=0.0573 t=2.645. n=3 mice/group.

- **f.** Primary microglia cultures were treated with LPS and IFNγ and assessed for expression of iNOS. A subset of cells were treated with Luteolin. One way ANOVA with Tukey's multiple comparisons test, a: P=0.0002, b: P=0.0002, c: P<0.0001, d: P<0.0001. ANOVA summary P<0.0001 F=55.75. n=3 independent litters.
- **g.** Mean number of oligodendrocyte lineage cells (SOX10+) per mm^2 ± s.e.m. in PBS or Luteolin treated GFAP-Nrf2 mice. 2-tailed Mann-Whitney test, P=0.5143. n=4 mice/group.
- **h.** Percentage of SOX10+ cells expressing Nrf2 per mm^2 ± s.e.m. in PBS or Luteolin treated GFAP-Nrf2 mice. 2-tailed unpaired Student's t- test, P=0.5193 t=0.7207. n=4 mice/group.
- **i.** Mean number of SOX10+ HMGCS1+ cells per mm^2 ± s.e.m. in PBS or Luteolin treated GFAP-Nrf2 mice. 2-tailed unpaired Student's t-test, P=0.6182 t=0.5303. n=4 mice/group (PBS), n=5 mice/group (Luteolin).
- **j.** Numbers of oligodendrocyte lineage cells (SOX10+) ± s.e.m. in primary cultures treated with Luteolin or DMSO vehicle control. 2-tailed unpaired Student's t-test, P=0.9049 t=0.1247. n=4 independent litters.
- **k.** Percentage of cultured SOX10+ cells ± s.e.m. which were MBP+ (light grey) or MBP- (dark grey). Two-way ANOVA with Bonferroni correction wild type vs GFAP-Nrf2, not significant. ANOVA summary (Interaction F(1,12)= 4.696, P-value=0.0511; Condition F(1,12)=0, P-value>0.9999; Cell types F(1,12)= 28158, P-value<0.0001). n=4 independent litters.
- **l.** Wildtype lesioned mice at 3 DPI following treatment with Luteolin or PBS from 0-3 DPI, immunostained for myelin debris using myelin basic protein (MBP). Scale bar, 100 μm.
- **m.** Percentage of lesion area covered by MBP myelin debris ± s.e.m. at 3 DPI in wildtype (WT) mice following treatment with Luteolin or PBS. 2-tailed unpaired Student's t-test, not significant P=0.3304 t=1.115. n=3 mice/group.
- **n.** Wildtype lesioned mice at 3 DPI following treatment with Luteolin or PBS from 0-3 DPI, immunostained for GFAP. Scale bar, 100 μm.
- **o.** Mean number of GFAP+ cells ± s.e.m. at 3 DPI in WT mice following treatment with Luteolin or PBS. 2-tailed unpaired Student's t-test, not significant P=0.9447 t=0.07441. n=3 mice/group.

  Source data is provided with this paper.

Supplementary Figure 17. Luteolin treatment of *Nfe2l2* conditional knockout in astrocytes.

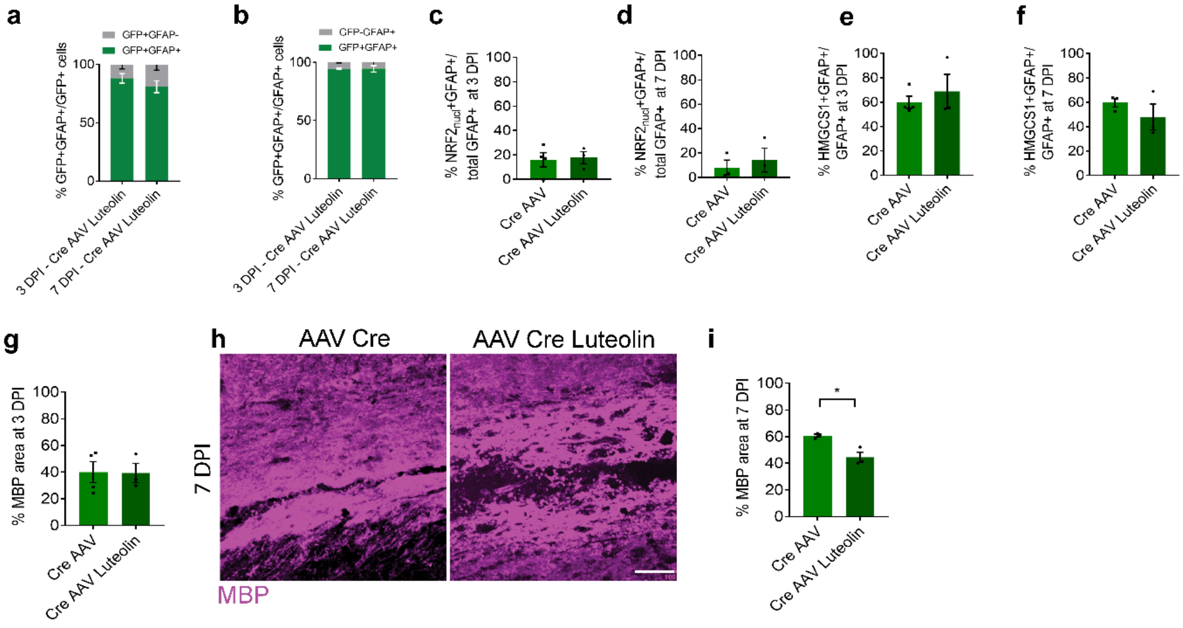

a. Mean percentage of GFP+ signal colocalized with GFAP in AAV-Cre + Luteolin treated mice at 3 and 7 DPI ± s.e.m.. n=3 mice per condition.

b. Mean percentage of GFAP+ cells expressing GFP in AAV-Cre + Luteolin treated mice at 3 and 7 DPI ± s.e.m.. n=3 mice per condition.

c. Mean percentage of GFAP+ cells with nuclear Nrf2 expression at 3 DPI in AAV-Cre ± Luteolin treated mice ± s.e.m.. 2-tailed unpaired Student's test, *P*=0.8314, t=0.02243. n=4 mice per condition (Cre AAV), n=3 mice per condition (Cre AAV Luteolin).

d. Mean percentage of GFAP+ cells with nuclear Nrf2 expression at 7 DPI in AAV-Cre ± Luteolin treated mice ± s.e.m.. 2-tailed Student's test, *P*=0.6235 t=0.531. n=3 mice per condition.

e. Mean percentage of GFAP+ cells expressing HMGCS1 at 3 DPI in AAV-Cre ± Luteolin treated mice ± s.e.m.. 2-tailed Mann-Whitney test, *P*>0.9999. n=3 mice per condition (Cre AAV), n=4 mice/group (Cre AAV Luteolin).

f. Mean percentage of GFAP+ cells expressing HMGCS1 at 7 DPI in AAV-Cre ± Luteolin treated mice ± s.e.m.. 2-tailed unpaired Student's test, *P*=0.3493 t=1.059. n=3 mice per condition.

g. Mean percentage area of MBP at 3 DPI (indicating demyelination) in AAV-Cre ± Luteolin treated mice ± s.e.m.. 2-tailed Student's test, *P*=0.9545 t=0.05997. n=3 mice per condition (Cre AAV Luteolin), n=4 mice/condition (Cre AAV).

h. MBP immunostaining at 7 DPI in AAV-Cre or AAV-Cre+Luteolin-treated mice. Scale bar, 100 µm.

i. Mean percentage area of MBP at 7 DPI (indicating remyelination) in AAV-Cre ± Luteolin treated mice ± s.e.m.. 2-tailed unpaired Student's t-test, *P* =0.0146 t=4.118. n=3 mice per condition.

Source data is provided with this paper.

Supplementary Figure 18. Summary Diagram.

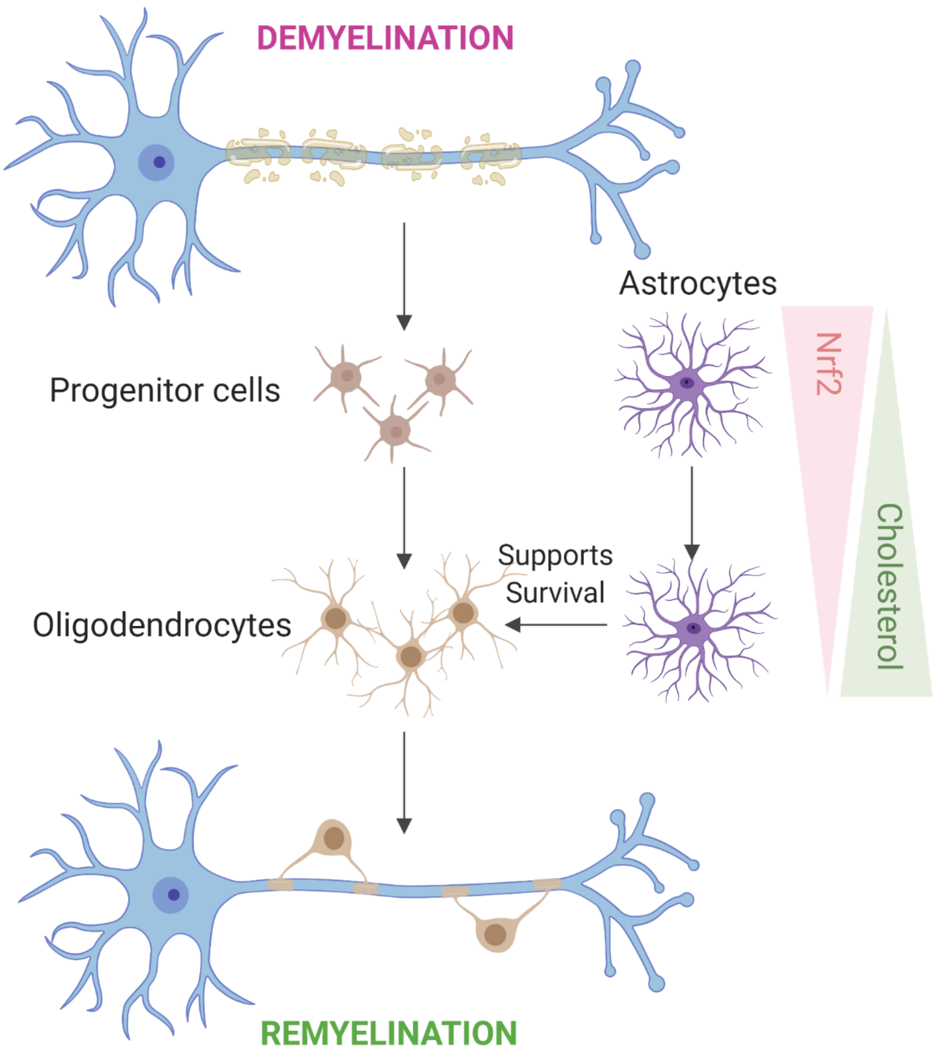

Following demyelination, astrocytes demonstrate transient activation of the Nrf2 pathway, which downregulates concomitant with upregulation of the cholesterol biosynthesis pathway, the latter associated with supporting survival of mature oligodendrocytes and subsequent efficient remyelination. Image created using Biorender.com.

Supplementary Table 1. Clinical information on human samples.
Clinical information on human brain tissue samples.MS=multiple sclerosis; SP= secondary progressive; PP= primary progressive; F=female; M=male; NA=not available; A=Active; I=Inactive; R=Remyelinated.

							Lesions		
	**ID**	**MS type**	**Sex**	**Age at death**	**Cause of death**	**Disease duration (years)**	**A**	**I**	**R**
**MS**	**MS121**	SP	F	49	MS	14	2	-	-
	**MS122**	SP	M	44	Bronchopneumonia	NA	1	2	-
	**MS100**	SP	M	46	NA	8	1	3	1
	**MS207**	SP	F	46	NA	25	1	2	2
	**MS176**	PP	M	37	Intestinal obstruction	27	-	1	3
	**MS136**	SP	M	40	NA	9	2	1	2
	**MS242**	SP	M	57	Sepsis	19	-	3	2
	**MS230**	SP	F	42	NA	19	3	-	-
**Control**	**CO14**	-	M	64	Cardiac failure	-	-	-	-
	**CO39**	-	M	82	Myelodysplastic syndrome, Rheumatoid arthritis	-	-	-	-
	**CO25**	-	M	35	Carcinoma of the tongue	-	-	-	-
	**CO28**	-	F	60	Ovarian cancer	-	-	-	-
