# Supplementary material for: Astrocyte-oligodendrocyte interaction regulates central nervous system regeneration
Source: Nat Commun. 2023 Jun 8;14:3372. doi: 10.1038/s41467-023-39046-8 (PMC10250470; doi:10.1038/s41467-023-39046-8)
Supplement: Supplementary file 3 — Description of Additional Supplementary Files [file 41467_2023_39046_MOESM3_ESM.pdf]

Description of Additional Supplementary Files

Supplementary Data 1:

TRAP sequencing of astrocytes during remyelination. Fragments per Kilobase Million (FPKM) and Log2 fold change of genes identified from TRAPseq of Aldh1l1-Rpl10- eGFP LPC-demyelinated lesions at 3, 7, and 10 days post injection (DPI) and no lesion controls. DE-Seq2 and Benjamini-Hochberg test, adjusted P values indicated.
